# Currents and Counter Currents in Medical Science

**Published:** 1860-07

**Authors:** 


*Currents and Counter Currents in Medical Science.*—The Annual Address to the Massachusetts Medical Society, by Dr. O. W. Holmes.

Dr. Holmes has rarely written anything which is not worthy a most attentive perusal. Clear, elegant and forcible in his style, he presents his wise and witty thoughts to us in an eminently attractive form. The pamphlet before us is no exception to the general character of the Doctor's works. It is a bold and somewhat radical statement of the doctrines of the *natura duce* school of medical reformers. Following in the footsteps of the celebrated Forbes, Dr. Holmes vehemently inveighs against the old system of pharmacolatry. Not unwisely did the old Greeks use the same word to denote a drug and a poison, and in this address the witty Doctor adopts and expounds the notion hidden under this curious old synonym. So broad and absolute, however, is his denunciation of polypharmacy, that his brethren in the Association were not prepared to follow him, and have cautiously prefixed to the pamphlet a copy of a resolution passed by them, disclaiming all responsibility for the sentiments of the address. We strongly commend the pamphlet to the notice of our readers.

